# LEAP4FNSSA lexicon: Towards a new dataset of keywords dealing with food security

**DOI:** 10.1016/j.dib.2022.108680

**Published:** 2022-10-17

**Authors:** Mathieu Roche, Agneta Lindsten, Tomas Lundén, Thierry Helmer

**Affiliations:** a CIRAD, F-34398 Montpellier, France; b TETIS, Univ. Montpellier, AgroParisTech, CIRAD, CNRS, INRAE, Montpellier 34090, France; c SLU University Library, Swedish University of Agricultural Sciences, 532 23 Skara, Sweden

**Keywords:** Food security, Semantic resources, Agrovoc, Text Mining

## Abstract

The main objective of the project LEAP4FNSSA (Long-term EU-AU Research and Innovation Partnership for Food and Nutrition Security and Sustainable Agriculture) is to provide a tool for European and African institutions to engage in a sustainable partnership platform for research and innovation on Food and Nutrition Security, and Sustainable Agriculture (FNSSA). The FNSSA roadmap facilitates the involvement of stakeholders for addressing and linking research to innovation dealing with food security issues. In this context, the LEAP4FNSSA project supports the driving of the roadmap. Research and innovation activities were captured in different data, i.e. LEAP4FNSSA database and heterogeneous textual data including project reports, websites, scientific publications, workshop reports and student theses. The Knowledge Extractor Pipeline System (KEOPS) was implemented to support the processing and analysis of textual data associated with FNSSA activities. KEOPS is based on the LEAP4FNSSA lexicon presented in this data paper. The LEAP4FNSSA lexicon composed of 331 keywords associated with 12 concepts of the food security domain is the result of 3 steps of work and brainstorming. The lexicon enables the capturing of research and innovation topics dealing with food security and conducted by African and European partners. This data paper presents the obtained lexicon and a summary of the method to build it.

**Specifications Table**

**Table:** 

Subject	Agricultural Sciences; Computer and Information Science; Social Sciences
Specific subject area	Lexicon in English dealing with food security
Type of data	Table
How data were acquired	Data are manually acquired by combining 3 types of resources (primary source): Pretoria vocabulary (obtained during a workshop organised in Pretoria in 2019), Agrovoc terms (https://www.fao.org/agrovoc/), and terms obtained by text mining. The LEAP4FNSSA lexicon is obtained with 3 iterative steps based on surveys and brainstorming with experts.
Data format	Filtered (LEAP4FNSSA lexicon) and raw (description of the process to build this lexicon).
Description of data collection	The dataset consists of (i) one table file with lexicon, (ii) a document describing the steps to obtain the final lexicon.
Data source location	The data are hosted on the CIRAD Dataverse. The data were built in the context of the LEAP4FNSSA project^1^.
Data accessibility	Repository name: CIRAD Dataverse. Data identification number: 10.18167/DVN1/D1C53L. Direct URL to data: https://www.doi.org/10.18167/DVN1/D1C53L

## Value of the Data

• This dataset contributes to the available resources for Natural Language Processing (NLP) and data mining on specialized domains and more precisely in the field of food security.
• This dataset is useful for computer scientists for enriching thesaurus and ontologies.
• This dataset can be used for indexing data bases (for instance these keywords could be proposed as metadata).
• This dataset can be used for analysing textual data dealing with agricultural sciences and social sciences.
• This list of keywords can be used as part of a search strategy protocol for systematic review research in areas related to food security.

## Data Description

In order to analyse textual data dealing with food security we have to consider different topics related to this issue. The proposed lexicon takes into account the multifactorial aspect related to food security with 331 keywords associated with 12 concepts summarized in Table 1. Examples of the concepts ”food security” and ”water management” are given in Tables 2 and 3. Note that both examples represent only 2 out of 12 concepts. All these concepts refer to different aspects of food security and sustainable agriculture in Africa and Europe. The 12 concepts are given in the Dataverse repository: https://doi.org/10.18167/DVN1/D1C53L.

**Table 1:** Number of keywords by concept.

Concept	Number of keywords	
Food security	20	
Agroecology	22	
Climate change	14	
Water management	37	
Crops	61	
Livestock and animal production	26	
One Health	34	
Agricultural intensification and innovation	31	
Food value chains and market	29	
Agricultural systems	20	
Partnerships in agricultural research development	11	
Research + Training	26	
**TOTAL**	**331**

**Table 2:** Keywords associated with the ”food security” concept.

food security	food access	food insecurity
household food security	food aid	food sovereignty
hunger	nutrition security	right to food
self-sufficiency	novel food	resource management
early warning	nutritional quality	malnutrition
socioeconomic sustainability	sustainable intensification	sustainable food security
urban nutrition security

**Table 3:** Keywords associated with the ”water management” concept.

water management	flood control	freshwater management
hydrological restoration	rain water management	water accounting
water auditing	water conservation	water extraction
water management in lowland	water management in upland	water security
water supply	water treatment	water conservation zone
drainage	hydraulic structure	water reuse
water storage	water use	agricultural hydraulics
watershed management	resource management	water resource
rural planning	water exploration	water rights
irrigation	groundwater storage	ground water storage
water quality	water governance	water harvesting
ict-based irrigation	drought	water constraint
hydrological monitoring		

## Experimental Design, Materials and Methods

The LEAP4FNSSA lexicon is the combination between 3 semantic resources, i.e. inputs in order to construct the final lexicon:

• *Pretoria vocabulary (list 1)*: This first lexicon composed of 8 concepts has been obtained during a workshop organised in Pretoria in 2019 in the context of the LEAP4FNSSA project. The process and the lexicon obtained are described in [1], [2]
• *Agrovoc vocabulary (list 2)*: Based on these 8 concepts (with one additional concept), Agrovoc terms associated with these concepts are manually extracted from the online^1^ resource. Agrovoc is a multilingual thesaurus dedicated to the agricultural domain developed by FAO (Food and Agriculture Organization) [3]. This thesaurus is used for different applications, e.g. indexing, annotation, data linking, etc.
• *Terms obtained by text-mining (list 3)*: Terminology is extracted from the LEAP4FNSSA corpus using generic parameters of the BioTex tool [4]. The LEAP4FNSSA corpus consists of documents and web pages relating to the FNSSA project database^2^. BioTex uses both statistical and linguistic information to extract terminology from free texts. The process applied is described in [5].

The initial terms (i.e. Pretoria vocabulary, Agrovoc vocabulary, terms obtained by text-mining) are given in the document ’LEAP4FNSSA_LEXICON_method_v2.pdf’ available in the Dataverse repository: https://doi.org/10.18167/DVN1/D1C53L.

The LEAP4FNSSA lexicon is obtained with 3 iterative steps. In these different steps, 4 types of experts and skills were involved: research scientist in text mining^3^, IT engineer^4^, experts in database indexing^5^, experts in food security issues (i.e. members of the LEAP4FNSSA project).

1. The first step based on the three inputs (i.e. *lists 1, 2 and 3*) involves the actions summarized below:
  • Starting point: the Agrovoc vocabulary (i.e. *list 2*) with 9 initial concepts and terms associated with FNSSA.
  • Based on a survey dedicated to Work Package 3 members of the LEAP4FNSSA project (10 answers), a term associated with 2 or more irrelevant labels is removed (strict pruning). Irrelevant labels are assigned by the LEAP4FNSSA members according to the point-of-view of their work and expertise.
  • For each concept, the Pretoria terms (i.e. *list 1*) are added to obtain a new lexicon.
  • The irrelevant terms of this new lexicon (based on a survey with 12 answers) are removed (strict pruning applied).
  • New terms proposed from surveys and brainstorming are added (i.e. LEAP4FNSSA workshop).
  • Selection of terms extracted by text-mining (i.e. *list 3*) labeled as relevant by Work Package 3 members (via a survey with 5 answers).
  • Final suggestions from the surveys are taken into account (e.g. remarks, new concepts, concepts to delete).
2. The second step is based on the following process:
  • Starting point: the lexicon obtained at step 1.
  • Improvement of concepts:
    • The ’Project management’ concept is deleted because this concept is not a major focus of the LEAP4FNSSA project and food security issues.
    • The ’Agroecology’ concept is added with terms proposed by Work Package 3 members.
  • Improvement of terms:
    • Terms are manually lemmatized.
    • Animals are added in the ’Agriculture and animal production’ concept.
    • Diseases are added in the ’One Health’ concept.
3. The last step is summarized below:
  • Starting point: the lexicon obtained at step 2.
  • Improvement of concepts:
    • Names of specific concepts have been changed.
    • Two new concepts are added: ’Food value chains and market’ and ’Agricultural systems’. These concepts contain new terms and terms that come from other concepts.
  • Improvement of terms:
    • New keywords are added after a work conducted by the experts in charge of data indexing of the FNSSA project database. For instance, keywords extracted from the FNSSA project database and manually validated by the experts are added.
    • Some terms are swapped between different concepts.
    • Ambiguous terms are deleted (e.g. capacity, agriculture, etc.)
    • The word ’crop’ is deleted in the 2-word terms of the ’Crops’ concept.
    • New keywords are integrated after a final checking by the experts in charge of data indexing.

These modifications to consolidate the LEAP4FNSSA lexicon (e.g. addition and/or deletion of concepts and/or terms) are detailed in the document ‘LEAP4FNSSA_LEXICON_method_v2.pdf ’.

Note that variations of terms could be automatically extracted with NLP approaches in dedicated corpora [6], [7]. This will be integrated as future work to extend the current lexicon.

The LEAP4FNSSA lexicon obtained is integrated into the KEOPS (Knowledge ExtractOr Pipeline System) tool that uses text mining approaches to highlight knowledge from heterogenous textual data [5]. KEOPS is currently implemented on LEAP4FNSSA data in order to extract, visualise and analyse food security themes with maps, graphs, curves, and Venn diagrams [8].

## Ethics Statement

No conflict of interest exists in this submission. The authors declare that the work described in this paper is original and not under consideration for publication elsewhere, in whole or in part. Its publication is approved by all the authors listed.

## CRediT authorship contribution statement

**Mathieu Roche:** Data curation, Methodology, Formal analysis, Writing – original draft. **Agneta Lindsten:** Data curation, Formal analysis, Writing – review & editing. **Tomas Lundén:** Data curation, Formal analysis, Writing – review & editing. **Thierry Helmer:** Data curation, Formal analysis, Writing – review & editing.

## Declaration of Competing Interest

The authors declare that they have no known competing financial interests or personal relationships that could have appeared to influence the work reported in this paper.

## Data Availability

LEAP4FNSSA lexicon (Original data) (Dataverse).

## Footnotes

3 first author

4 last author

5 second and third authors

## Footnotes

1 https://www.fao.org/agrovoc/

2 https://www.library.wur.nl/WebQuery/leap4fnssa-projects
